# The expression of FHIT, PCNA and EGFR in benign and malignant breast lesions

**DOI:** 10.1038/sj.bjc.6603512

**Published:** 2006-12-12

**Authors:** G Terry, L Ho, P Londesborough, C Duggan, A Hanby, J Cuzick

**Affiliations:** 1 Department of Epidemiology, Mathematics and Statistics, Cancer Research UK, Queen Mary University of London, Wolfson Institute, Charterhouse Square, London ECIM 6BQ, UK; 2 St. James's University Hospital, Beckett Street, Leeds LS9 7TF, UK

**Keywords:** FHIT, PCNA, EGFR, BBD, CIS, Ca

## Abstract

Immunohistochemical staining for FHIT and PCNA proteins was carried out in 451 breast lesions showing nonproliferative benign breast disease (BBD) (*n*=263), proliferative BBD without atypia (*n*=128), proliferative BBD with atypia (*n*=11), carcinoma *in situ* (*n*=15) or invasive carcinoma (*n*=34) and for EGFR protein in a subset of 71 of these cases. FHIT underexpression was not detected in nonproliferative lesions, but occurred in 2% of proliferative BBD without atypia, 10% proliferative BBD with atypia, 27% of carcinoma *in situ* and 41% of invasive carcinoma, which suggests that it could be useful in assessing those carcinoma *in situ* lesions (ductal, DCIS and lobular, LCIS) that are more likely to progress to malignancy. Preliminary microarray comparisons on DCIS and invasive carcinoma samples dissected from formalin-fixed paraffin sections showed a consistent downregulation of two previously identified FHIT-related genes, caspase 1 and BRCA1 in lesions underexpressing FHIT.

Benign breast disease (BBD) and carcinoma *in situ* (ductal, DCIS and lobular, LCIS) represent a group of histologically heterogeneous lesions, some of which are associated with increased risk for invasive breast cancer. In moderate to florid usual type ductal hyperplasia (UDH) and in papillomas, the relative risk (RR) of developing invasive breast cancer is small (RR=1.5–2.0), but this rises in atypical ductal hyperplasia (ADH, RR=4.0–5.0) (Page and Dupont, 1989) and in carcinoma *in situ* (DCIS or LCIS, RR=8.0–10.0) (Page, 1991; Page *et al*, 2000). Pathological and clinical evidence suggests that different BBD lesions have different magnitudes of risk for the subsequent development of malignancy and one of the key challenges is to identify at an early stage those BBD lesions which could progress to cancer. Certain pathologies have been associated with higher risk of malignancy, for example atypical ductal hyperplasia (ADH) and LCIS, but their diagnosis is subject to considerable interobserver variability (Elston *et al*, 2000) and therefore any independent molecular attribute could help in improving consistency as well as providing insight into the underlying biology.

Underexpression of the *FHIT* gene has frequently been linked to human cancer including breast cancer (Pekarsky *et al*, 2002). The *FHIT* gene is located at chromosome 3p14.2. It spans 1.8 Mb and has 10 exons. Exons 5–9 code for a small mRNA of 1.1 kb, which is susceptible to modification by alternative splicing and downregulation by promoter methylation. The *FHIT* protein (16.8 kDa) is mainly localised in the cytoplasm of epithelial cells complexed with tubulin and a ubiquitin conjugating enzyme, *UBC9* (Shi *et al*, 2000; Golebiowski *et al*, 2004). Experimental results have shown that *FHIT* protein functions as a hydrolase for intracellular diadenosine triphosphate that is involved in the control of cell growth. It is associated with check point proteins *ATR* and *CHK1* (Hu *et al*, 2005) and is a target for Src protein kinase, suggesting that *FHIT* protein is also involved more directly in the cell cycle (Pekarsky *et al*, 2004). In addition, *FHIT* is known to be a proapoptotic protein closely associated with *FADD*, *caspase-8* (Dumon *et al*, 2001; Ishii *et al*, 2001; Roz *et al*, 2002, 2004), *MDM2* and *p53* (Nishizaki *et al*, 2004).

In the breast, loss of heterozygosity (LOH) at the *FHIT* locus has been observed in 45% of invasive cancers (Ca) and in 11% of unspecified preneoplastic lesions including usual type ductal hyperplasia (UDH), aprocrine metaplasia, DCIS and intraductal papilloma (Maitra *et al*, 2001). In another study, reduction or loss of *FHIT* protein expression was found in 40–80% of Ca and in 60% of CIS (Gatalica *et al*, 2000; Yang *et al*, 2001). Univariate analysis of disease-free survival showed *FHIT* to be a significant prognostic factor in patients with early breast cancer (Yang *et al*, 2001; Ginestier *et al*, 2003). Although the frequency of abnormalities in the *FHIT* gene is comparable to, if not in excess of those found for currently used biomarkers for breast cancer such as *p53* or *HER2* (Stark *et al*, 2000; Arun *et al*, 2005), the potential role for *FHIT* in predicting malignant progression in BBD has not been fully assessed. We report here the expression of *FHIT* and *PCNA* proteins in 451 cases with benign or malignant breast disease and *EGFR* in a subset of 71 of these cases. Our results show that underexpression of *FHIT* may have some use as a marker of breast disease progression in BBD. As the incidence of *FHIT* underexpression increases at the stage of carcinoma *in situ*, we have undertaken a preliminary examination of the cell environment associated with normal and abnormal *FHIT* expression in DCIS and invasive carcinoma using expression microarrays to evaluate the possible function of this protein in breast tissues. The use of *FHIT* protein detection for the monitoring of breast disease progression is discussed.

## MATERIALS AND METHODS

Formalin-fixed and paraffin-embedded (FFPE) sections were obtained from 787 archival blocks from 532 women. Biopsies from 451 women were tested and reported in this study, made up of the following groups: nonproliferative BBD (*n*=263), proliferative BBD without atypia (*n*=128), proliferative BBD with atypia (*n*=11), carcinoma *in situ* (*n*=15) and invasive carcinoma (*n*=34). Biopsies from 10 women with incomplete histology data and from 71 women whose tissue sections were damaged during staining were excluded from the study. The routine and reviewed histology results were combined. To classify the biopsy and select a lesion from several co-existing lesions, preference was given to lesion categories in the order of severity: Invasive carcinoma >carcinoma *in situ* >proliferative lesions with atypia >proliferative lesions without atypia >nonproliferative lesions (Page *et al*, 2000). Among coexisting lesions within the same category, preference was given in the order of : extent >severity.

Sections for immunohistochemical staining (IHS) were 3 μm thick and were stored at 4°C. Sections for microarray analysis were 10 μm thick and stored at −80°C. Ethics approval was obtained from Guy's Hospital NHS Trust Ethics Committee for the use of this archival material.

### Immunohistochemical staining

All sections were de-waxed, processed for epitope retrieval and stained as previously described (Terry *et al*, 2004). Rabbit anti-FHIT antibody (Zymed Laboratories Inc., California, USA) and horse-radish peroxidase (HRP)-conjugated anti-rabbit antibody (Abcam Ltd, Cambridgeshire, UK) were used for detection of FHIT protein. Mouse anti-*PCNA* antibody (Sigma-Aldrid Ltd, Dorset, UK) and alkaline phosphatase (ALP)-conjugated goat anti-mouse (Abcam) were used for *PCNA* protein. *FHIT* and *PCNA* proteins were stained simultaneously. Mouse anti-*EGFR* antibody (Abcam) and HRP-conjugated anti-mouse antibody (Abcam) were used for the detection of *EGFR* protein, with haematoxylin as counter-stain. HRP and ALP were detected using the corresponding detection kits (Abcam).

The results were scored ‘blind’ and independently by three laboratory scientists. A lesion was scored positive if the stained section from any of the associated paraffin blocks was scored positive by two or more observers. The scoring system used for each protein was as follows:

- *FHIT*. Cytoplasmic staining was graded as normal (↔) or underexpressed (↓). Underexpression was assigned if >30% of cells in the designated histological category showed absent or reduced staining when compared to adjacent normal breast tissue.
- *PCNA*. Nuclear staining of 500 nuclei in the designated histological category were counted. Overexpression (↑) was assigned if >10% of the nuclei showed either (i) red trabeculated, (ii) intensely red or (iii) cloudy red stain. Otherwise, the lesion was scored as normal (↔).
- *EGFR*. Membrane staining for *EGFR*. Overexpression (↑) was assigned if complete membrane staining was present in the designated histological category. Sections obtained from lesions, which showed *EGFR* gene amplification, was used as control. Lesions with no staining were scored as normal (↔). Of the 79 stained lesions, eight (two with *FHIT* underexpression and six with normal *FHIT* expression) showed either cytoplasmic staining only or cytoplasmic and membrane staining combined and were excluded from the analysis. Intense cytoplasmic stain in some cells can lead to a false impression of a positively stained membrane.

### *EGFR* gene amplification analysis by polymerase chain reaction

DNA extracts from 14 available paraffin sections were analysed. Quantitation of polymerase chain reaction (PCR) fragments was carried out by AlfExpress as previously described (Terry *et al*, 2004). The primers used were as follows:

- *EGFR* exon 8 forward 5′-CGCAAGTGTAAGAAGTGCGAA-3′, reverse 5′-CGTAGCATTTATGGAGAGTGAGTCT-3′.
- *GAPDH* exon 9 forward 5′-CCCCCACCACACTGAATCT-3′, reverse 5′-CTAGGCCCCTCCCCTCTT-3′.

### Microarray analysis using human cancer arrays (MWG Biotech, Ebensberg, Germany)

- RNA extraction and amplification (1.5 cycles) from paraffin-embedded sections were carried out using the Paradise Reagent System from Arcturus Bioscience Inc., California, USA, in accordance with the manufacturer's instruction.
- Incorporation of Cy3- or Cy5-UTP (Perkin-Elmer Life and Analytical Sciences) was carried out during the IVT stage of the final amplification half-cycle using MEGAscript reagents (Ambion Inc., Cambridgeshire, UK) in accordance with the manufacturer's instructions and the amplified RNA (aRNA) purified using Arcturus Paradise kit reagents.
- aRNA analysis. aRNA was quantitated spectroscopically using an ND-1000 spectrophotometer (NanoDrop Technologies, Delaware, USA) and its size measured by electrophoresis in formaldehyde-containing agarose gels (Maniatis *et al*, 1982). In total, 15 *μ*g each of Cy3- and Cy5-labelled aRNAs were mixed and used for hybridisation to each array.
- Hybridisation to MWG human cancer gene arrays spotted with 50-mer oligonucleotides specific for 1853 human genes involved in cancer development was carried out according to the manufacturer's protocol (MWG Biotech, Ebersberg, Germany). Arrays were scanned by MWG Scanning Service (Ebersberg, Germany) using an Affymetrics 428 scanner. Each channel (Cy3 or Cy5) was scanned at 10 *μ*m resolution at three different photomultiplier gain settings. Fluorescence intensity values from each channel were processed using Imagene 4.2 software (Biodiscovery, Inc., California, USA). To obtain maximal signal intensities without saturation effects, intensity values from Tiff images were integrated into one value per probe by the MAVI software (Version Pro 2.6.0, MWG Biotech, Ebersberg, Germany). A negative control threshold was calculated from the mean fluorescence intensities obtained for 28 control *Arabidopsis* oligonucleotides. Fluorescence intensity values for cellular genes that exceeded this threshold by two standard deviations were regarded as significant expression signals.

Analysis of the signals was carried out by MWG Analysis Service (Ebersberg, Germany). Essentially, signals were calculated as median intensity minus median background. Sample intensities were normalised by the 50th percentile (median) method using all of the spot-filtered genes. Spots flagged bad or not found were excluded from further analysis.

Analysis of trend and variance was carried out using STATA Statistics and Data Analysis package v 8.2.

## RESULTS

### Staining for *FHIT*, *PCNA* and *EGFR*

**Figure 1:**
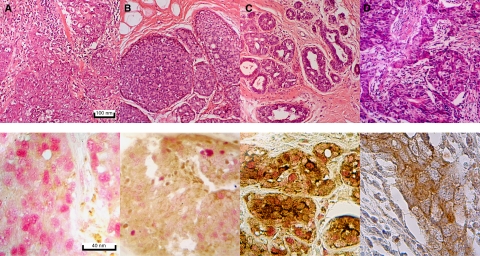
Staining patterns in four breast lesions. Upper row (H&E). Cancer (**A** and **D**), DCIS (**B**) and hyperplasia (**C**). Lower row (ISH). Cancer (**A**), DCIS (**B**) and hyperplasia (**C**) dually stained with antibodies to FHIT (brown) and PCNA (red). (**D**) Cancer stained with antibody to EGFR (brown) and haematoxylin (blue). Arrows indicate different staining patterns: PCNA (i) trabeculated, (ii) intense, (iii) cloudy, (iv) cloudy and EGFR (v) membrane-associated.

Figure 1 shows H&E and dual staining for *FHIT* and *PCNA* proteins of (A) an *FHIT*-negative cancer, (B) an *FHIT*-reduced low-grade *DCIS* and (C) an *FHIT*-positive florid hyperplasia. A cancer stained with H&E and *EGFR* is shown in (D). Arrows indicate *PCNA*-positive trabeculated staining, *PCNA*-positive intensive staining and *PCNA*-positive cloudy staining nuclei.

### The expression of *FHIT*, *PCNA* and *EGFR* proteins in breast lesions

**Figure 2:**
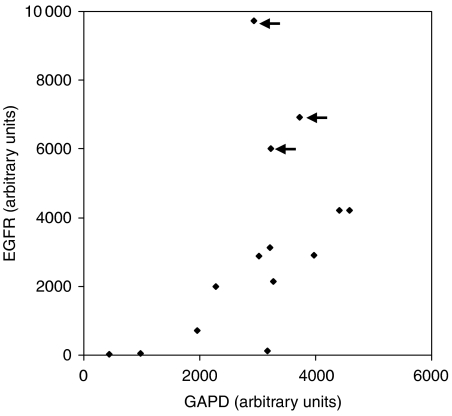
The detection of FHIT, PCNA and EGFR expression in nonproliferative and proliferative breast lesions.

**Figure 3:**
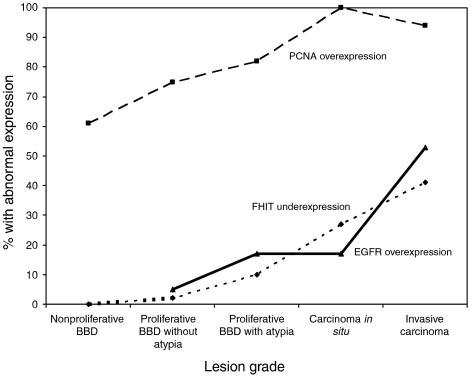
Quantitation of Cy5-labelled EGFR and GAPDH amplicons (in arbitrary units) from 14 EGFR-positive lesions by sequencing gel electrophoresis (AlfExpress) using Fragment Manager v.1.2 software (Pharmacia). ←, gene amplification.

The expression of *FHIT* and *PCNA* in all 451 biopsies and EGFR in a subset of 71 biopsies showing proliferative changes is summarised in Table 1 and Figure 2. *FHIT* was expressed normally in all nonproliferative BBD lesions, but was underexpressed in 12% of all proliferative lesions particularly in those with atypia and cancerous changes (χ^2^_trend_=114.31, *P*=<0.0001). In all, 60–100% of all lesions overexpressed *PCNA* and 5–53% overexpressed *EGFR* in parallel with increasing lesion grades (χ^2^_trend_=13.02, *P*=0.00031). Only three of the 14 cases overexpressing *EGFR* showed *EGFR* gene amplification relative to the *GAPDH* housekeeping gene (Figure 3). All lesions, which underexpressed *FHIT*, also overexpressed *PCNA* (Table 2). Although *FHIT* underexpression and *EGFR* overexpression followed a overall similar distribution in proliferative BBD lesions and in invasive carcinomas, they were not always concordant in individual cases. Three DCIS and seven invasive carcinomas with normal *EGFR* expression underexpressed *FHIT*, whereas two DCIS and 11 invasive carcinomas with normal *FHIT* expression overexpressed *EGFR* (Table 2). Multivariate analysis showed correlation between the three markers together and increasing lesion grades (*F*=5.582, *P*=0.0017).

### Comparison of *FHIT*-related gene expression in ductal carcinoma *in situ* and cancer lesions

RNA extracted from paired *FHIT*-positive and *FHIT*-negative lesions were amplified, labelled separately with either Cy3- or Cy5-UTP and pooled. The amounts of aRNA recovered after two amplification cycles was 16–20 *μ*g and the predominant size was 300–600 bases, but extending up to 2000 bases (data not shown). Differentially labelled RNA from three DCIS pairs and one invasive carcinoma pair were hybridised to separate human cancer gene arrays.

**Figure 4:**
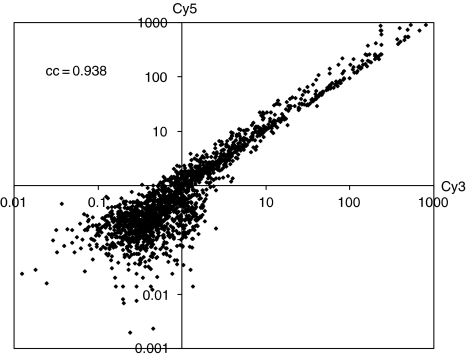
Correlation of Cy3-labelled FHIT-positive DCIS and Cy5-labelled FHIT reduced DCIS hybridised to MWG human cancer gene array.

Figure 4 shows good pair-wise correlation between RNA extracted from an *FHIT*-positive DCIS labelled with Cy-3 and an *FHIT*-negative DCIS labelled with Cy-5 hybridised to 1853 cancer gene probes with a correlation coefficient of 0.938. A comparison of the Cy3/Cy5 ratios showed that two genes, *caspase 1* and *BRCA1*, were underexpressed in all four lesions with reduced *FHIT* expression (Table 3).

## DISCUSSION

Oncogenesis represents an interplay between cell proliferation and apoptosis. Markers for these biological activities have been extensively sought in breast cancer for prognosis, prediction of treatment effectiveness and development of new chemotherapeutic agents. Whether the same markers can be used for predicting risk of subsequent changes in BBD and precursor progression in DCIS has not been extensively studied. We have used the cell cycle protein *PCNA* and the mitogenic receptor *EGFR* as markers of proliferation, and *FHIT* as a marker associated with apoptosis. Table 1 and Figure 2 summarise the relationship between the proliferative activities observed in breast lesions and *PCNA*, *EGFR* or *FHIT* expression. Of the 451 cases studied, 312 (69%) were found to be positive for *PCNA*. The positivity rate is comparable to that reported previously (Steck and El-Naggar 1994; Fabian *et al*, 2002; Honrado *et al*, 2005). The synthesis of *PCNA* is closely associated with the normal G1/S transition of the cell cycle and the protein has a comparatively long half-life. It is, therefore, a good marker for measuring proliferative activity in breast lesions where cells are at different phases of growth and accounts for its apparent expression in 94–100% of *in situ* and invasive carcinomas and even 61% of histologically ‘nonproliferative’ BBD (Table 1, Figure 2). In contrast, overexpression of the mitogenic signalling mediator *EGFR*, indicative of abnormalities in the commitment of G1 to S phase in the cell cycle, was found in 30% of breast lesions, predominantly in invasive carcinoma (53%). Similar rates of *EGFR* overexpression have been reported previously in DCIS and invasive carcinoma (Knoop *et al*, 2001; Lebeau *et al*, 2003). The causes of *EGFR* overexpression are not well understood but aneusomy of chromosome 7 on which the *EGFR* gene is located does not relate directly to *EGFR* protein overexpression (Bhargava *et al*, 2005; Sauer *et al*, 2005). In our study, gene amplification could only account for three out of 14 cases (Table 1, Figure 3), but our method would not distinguish between aneusomy of chromosome 7 from any other mechanism for gene amplification.

Overall, our results suggest that most BBD lesions and carcinomas contain populations of rapidly dividing normal and/or abnormal cells and both are predisposed to accumulation of genomic changes. This could include the establishment of *FHIT* underexpression in a subset of proliferative BBD lesions and carcinomas.

*FHIT* underexpression was found in 14 out of 34 (41%) of invasive carcinomas and in four out of 12 (33%) of DCIS (Table 1, Figure 2). Previously, loss of *FHIT* expression in cancer has been found to mirror other poor prognosis markers, being positively correlated with *Bcl-2* and *p53* overexpression, proliferative activity and aggressive histological phenotype and negatively correlated with oestrogen and progesterone receptor status (Campiglio *et al*, 1999; Yang *et al*, 2001; Arun *et al*, 2005). Loss of *FHIT* expression has also been found to confer a significant disadvantage in disease-free survival (Yang *et al*, 2001; Ginestier *et al*, 2003). However, the incidence of *FHIT* expression in BBD lesions in the absence of invasive carcinoma was unclear. In a study involving 50 cancers, Gatalica *et al* (2000) found reduced *FHIT* protein expression in four out of 34 (12%) hyperplastic epithelium (grades not specified), 26 out of 47 (55%) atypical hyperplasia and DCIS combined and 33 out of 46 (72%) cancers. In a study of 45 cancers, LOH at the *FHIT* locus 3p14.2 was found in five out of 45 (11%) of cancer-associated pre-neoplasia (grades not specified but included UDH, apocrine metaplasia, DCIS and intraductal papilloma) and 20 out of 44 (45%) cancers (Maitra *et al*, 2001). In both studies, the BBD lesions examined were adjacent to cancer tissues and the staining characteristics observed may not be a reflection of the true *FHIT* status as this could have arisen from a local field effect (Cavalli *et al*, 2004). In our study, each of the cases tested was classified by the highest grade of lesion present. We found normal expression of *FHIT* protein in all nonproliferative lesions, but underexpression in 2% of proliferative lesions without atypia, 10% of proliferative lesions with atypia, 0% of lobular carcinoma *in situ*, 33% of ductal carcinoma *in situ* and 41% of invasive carcinomas (Table 1). The outcome was known for eight of the 12 DCIS lesions. Two of three lesions with *FHIT* underexpression and five of five lesions with normal *FHIT* expression progressed subsequently to invasive breast cancer, but the difference was not significant (*P*=0.375). Similarly, no relationship could be ascertained between *FHIT* underexpression and different pathological types of *in situ* or invasive carcinoma because of the small number of lesions analysed.

*In vitro* experiments have shown that *FHIT* is a proapoptotic protein which operates via both the extrinsic (Roz *et al*, 2004) and intrinsic pathways (Dumon *et al*, 2001; Ishii *et al*, 2001). In a study involving 100 colorectal adenocarcinomas, Mady and Melhem (2002) found that overexpression of *FHIT* is directly proportional to the rate of apoptosis. It was therefore of interest to assess if underexpression of *FHIT* in our biopsies is likely to have any functional effect. To do this, we used expression microarray analysis to assess the expression levels of other apoptosis-associated and breast cancer prognostic genes (Table 3). A commercially available protocol (Paradise kit, Arcturus, Biosciences Inc., California, USA) was successful in amplifying RNA retrieved from formalin-fixed and paraffin-embedded breast lesions. Pair-wise analysis confirmed other reports (Ma *et al*, 2003; True *et al*, 2006) that good-quality aRNA could be reproducibly obtained for hybridisation to microarrays (Figure 4). This technique is invaluable for analysis of archival material. Although only three of the four DCIS lesions with underexpressed *FHIT* expression (Table 1) were available for testing in this study, consistent downregulation of two genes, namely *caspase 1* and *BRCA1*, was noted. *Caspase 1* is an important regulator of epithelial cell apoptosis and its downregulation has been reported in breast, gastric, colon and prostate cancers (Boudreau *et al*, 1995; Jarry *et al*, 1999; Winter *et al*, 2001; Jee *et al*, 2005). Loss of *BRCA1* is associated with a more aggressive phenotype in sporadic breast cancer (Jarvis *et al*, 1998; Taylor *et al*, 1998) and concomitant loss of *FHIT* and *BRCA1* alleles has also been reported in a number of repair-deficient cancers including breast cancer and ovarian cancers (Wilson *et al*, 1999; Turner *et al*, 2002; Santos *et al*, 2004).

In this study, coexpression of *PCNA* and *FHIT* was determined in the same cell population by dual staining, and *EGFR* was monitored in the same cell population in an adjacent section. This allows a simultaneous assessment of an interplay between the expression of the three proteins. Our results showed that the presence of *PCNA* does not clearly distinguish between nonproliferative BBD lesions and proliferative BBD lesions (with or without atypia), or between *in situ* carcinomas and invasive carcinomas (Table 1). In contrast, underexpression of *FHIT* was associated with lesions with increasing severity (*χ*^2^_trend_=114.31), including four out of 12 DCIS and 14 out of 34 invasive carcinomas. Three DCIS and seven invasive carcinomas with normal *EGFR* expression underexpressed FHIT (Table 2), which suggest that detection of *FHIT* expression could be of use either alone or with other markers such as *EGFR*, in identifying a subset of proliferative breast lesions with malignant potential. In contrast to the current lack of consensus in scoring *EGFR* expression by IHS, detection of *FHIT* is technically simple and the incorporation of a quantitation step by image analyser would provide an objective measure of its expression (Mady and Melhem, 2002). The loss of *caspase 1* in DCIS lesions also merits further analysis since the effectiveness of a number of therapeutic drugs depends on its activation.

## Figures and Tables

**Table 1:** FHIT, PCNA and EGFR proteins in nonproliferative and proliferative breast lesions

**Pathology**			**Relative risk of cancer (**Page *et al*, 2000)	**Breast lesions**	**No. of cases with expression status as**					
**Lesion**	**Category (**Stark *et al*, 2000)				**FHIT**		**PCNA**		**EGFR**	
					**↓**	**↔**	**↑**	**↓**	**↑**	**↔**
BBD	(a) Nonproliferative		1.0	Normal	0	60	24	36	ND	ND
				Inflammation	0	13	6	7	ND	ND
				Cyst	0	4	3	1	ND	ND
				Metaplasia	0	18	15	3	ND	ND
				Blind duct adenosis	0	60	35	25	ND	ND
				Hyperplasia (usual type)	0	24	17	7	ND	ND
				Fibroadenoma	0	84	60	24	ND	ND
		Subtotal			0 (0%)	263	160 (61%)	103	NA	NA
										
	(b) Proliferative without atypia		1.5–2.0	Papilloma	0	25	16	9	ND	ND
				Sclerosing adenosis	0	36	29	7	ND	ND
				Hyperplasia (moderate)	1	47	35	13	0	4
				Hyperplasia (florid)	2	17	16	3	1^a^	14
		Subtotal			3 (2%)	125	96 (75%)	32	1 (5%)	18
										
	(c) Proliferative with atypia		4.0–5.0	ADH/ALH	1 (10%)	10	9 (82%)	2	1 (17%)	5
										
Carcinoma	(a) *In situ*		8.0–10.0	LCIS	0	3	12	0	0	3
				DCIS	4	8	3	0	2	9
		Subtotal			4 (27%)	11	15 (100%)	0	2 (17%)	12
										
	(b) Invasive		NA		14 (41%)	20	32 (94%)	2	17^b^(53%)	15
										
					22(5%)	429	312 (69%)	139	21 (30%)	50
										
Total					*n*=451		*n*=451		*n*=71	
										

a Gene amplification detected in one case; ↔=normal level of expression; ↑=overexpression; ↓=underexpression; ND=not done; NA=not applicable.

b Gene amplification detected in 2 case.

**Table 2:** Corrleation of FHIT, PCNA and EGFR expression in nonproliferative and proliferative breast lesions

**Category**	**Grade**		**Expression status (no. of cases)**									
			**FHIT**	**↔**	**↔**	**↓**	**↓**	**FHIT**	**↔**	**↓**	**↓**	**↔**
			**PCNA**	**↑**	**↓**	**↑**	**↓**					
								**EGFR**	**↑**	**↑**	**↔**	**↔**
BBD	(a) Nonproliferative	Normal		24	36	0	0		ND	ND	ND	ND
		Inflammation		6	7	0	0		ND	ND	ND	ND
		Cyst		3	1	0	0		ND	ND	ND	ND
		Metaplasia		14	4	0	0		ND	ND	ND	ND
		Blind duct adenosis		35	25	0	0		ND	ND	ND	ND
		Hyperplasia (usual type)		17	7	0	0		ND	ND	ND	ND
		Fibroadenoma		60	24	0	0		ND	ND	ND	ND
												
	(b) Proliferative	Papilloma		16	9	0	0		ND	ND	ND	ND
	without atypia	Sclerosing adenosis		29	7	0	0		ND	ND	ND	ND
		Hyperplasia (moderate)		34	13	1	0		0	0	1	3
		Hyperplasia (florid)		14	3	2	0		1	0	0	14
												
	(c) Proliferative	ADH/ALH		8	2	1	0		0	1	0	5
	with atypia											
												
Carcinoma	(a) *In situ*	LCIS		3	0	0	0		0	0	0	3
		DCIS		8	0	4	0		2	0	3	6
												
	(b) Invasive			18	2	14	0		11	6	7	8
												
Total				289	140	22	0		14	7	11	39
				*n*=451		*n*=71						

↔=normal level of expression; ↑=overexpression; ↓=underexpression; ND=not done.

**Table 3:** Cy3/Cy5 ratio in FHIT and/or breast cancer related genes

**Association**	**Genes**	**Cy3/Cy5 ratio**			
		**Ca**	**DCIS**		
		**F+/F−**	**F+/F±**	**F+/F±**	**F+/F±**
	Median value for all genes	1.041/0.789	0.753/0.456	1.048/0.690	0.979/0.647
	Median ratio for all genes	1.319	1.651	1.519	1.513
					
FHIT-apoptosis	APAF1	0.022	1.228	0.528	0.698
	BAD	0.394	1.530	0.737	0.629
	BAX	3.481	0.882	1.322	1.078
	BCL2 (probe 1)	0.973	0.649	0.744	0.912
	BCL2 (probe 2)	0.340	2.053	1.028	0.638
	BIRC5	0.296	2.335	1.170	0.453
	CASP1	1.666	2.263	8.992	4.758
	CASP10	2.089	0.535	0.716	1.273
	CASP8	2.998	1.035	0.940	0.939
	CDC2	ND	ND	1.758	0.337
	COX7A2L	2.637	1.989	1.462	1.060
	CFLAR	0.627	1.172	0.988	0.903
	FADD	0.180	1.900	1.167	0.616
	FAS	0.685	1.044	3.079	4.253
	FASLG	0.114	0.578	1.213	0.641
	IL1B	0.030	0.554	1.058	0.660
	IL1RN	0.039	0.732	0.679	1.697
					
FHIT-cell cycle	ATM	0.351	0.772	1.111	0.776
	WWOX	3.112	0.902	1.122	1.185
					
Breast cancer prognostic markers	AKT1	1.254	0.734	0.759	0.850
	AR	0.310	0.780	1.016	0.646
	BRCA1 (probe 1)	0.660	1.527	1.085	1.650
	BRCA1 (probe 2)	1.724	14.354	13.737	23.800
	BRCA2	1.537	ND	1.267	1.338
	EGFR	2.858	0.527	1.318	0.998
	ERBB2	1.975	0.751	0.617	0.520
	ERBB3	0.683	1.539	0.863	3.632
	ERBB4	2.036	0.980	0.808	1.064
	ESR1	0.415	6.568	0.970	0.551
	MLH1	0.089	0.869	2.323	0.952
	MLH3	2.134	0.646	2.710	24.352
	PCNA	0.236	0.630	0.801	0.526
	PGR	0.991	1.737	20.092	10.050
	SRC	0.135	0.710	1.515	0.889
	TNF	1.583	0.934	0.684	0.708
	TP53	0.933	5.030	0.689	0.607

ND=instensity in one or both channels below the cutoff levels set by the MAVI software; F+=normal FHIT expression; F− or F±=FHIT underexpression.
